# *Factor V* G1691A is associated with an increased risk of retinal vein occlusion: a meta-analysis

**DOI:** 10.18632/oncotarget.20636

**Published:** 2017-09-04

**Authors:** Yuanyuan Zou, Xi Zhang, Jingyi Zhang, Xiangning Ji, Yuqing Liu

**Affiliations:** ^1^ The Second Department of Ophthalmology, Cangzhou Central Hospital, 061001, Cangzhou, PR China

**Keywords:** factor V, retinal vein occlusion, polymorphism, meta-analysis

## Abstract

We performed a meta-analysis to investigate the association between the Factor V G1691A polymorphism and the risk of retinal vein occlusion (RVO). This analysis included 37 studies involving 2,510 cases and 3,466 controls. Factor V G1691A was associated with an increased risk of RVO in the allele, heterozygote, dominant, and carrier models (*P*A < 0.001, odds ratios >1), but not the homozygote or recessive models (*P*A > 0.05). Similar results were observed in a meta-analysis of central retinal vein occlusion (CRVO) and when comparing Caucasian subgroups to population-based controls. These data demonstrate that the G/A genotype of Factor V G1691A is associated with an increased risk of RVO/CRVO in a Caucasian population.

## INTRODUCTION

Retinal vein occlusion (RVO) is a multifactorial vascular disease characterized by retinal blood stasis, venous tortuous expansion, retinal hemorrhage, and edema that can cause loss of visual acuity loss or blindness [1]. There are two main types of RVO, branch retinal vein occlusion (BRVO) and central retinal vein occlusion (CRVO), which are classified according to the sites of occlusion [2, 3]. Systemic vascular disorders including hypertension, arteriosclerosis, and diabetes mellitus, as well as genetic background and environmental factors have been associated with the risk of RVO [4, 5]. Single nucleotide polymorphisms (SNPs) in several hemostasis-associated genes such as *Factor V*, *Prothrombin* (*Factor II*), and *PAI-1*, may contribute to the pathogenesis of RVO [4, 6].

Factor V, a co-factor in the prothrombinase complex, has an essential role in blood coagulation, and modulates the conversion of prothrombin to thrombin [7]. *Factor V* G1691A (*Factor V* Leiden or R506Q), is a frequently observed mutation in *Factor V* that has been associated with activated protein C (APC) resistance and several diseases including Budd-Chiari syndrome, portal vein thrombosis, and RVO [8–10]. The most recent meta-analysis of genetic variants associated with RVO was published in 2013 and involved 21 case-control studies [11]. Therefore, we performed an updated meta-analysis of 37 case-control studies under all genetic models.

## RESULTS

### Identification of eligible case-control studies

**Figure 1:**
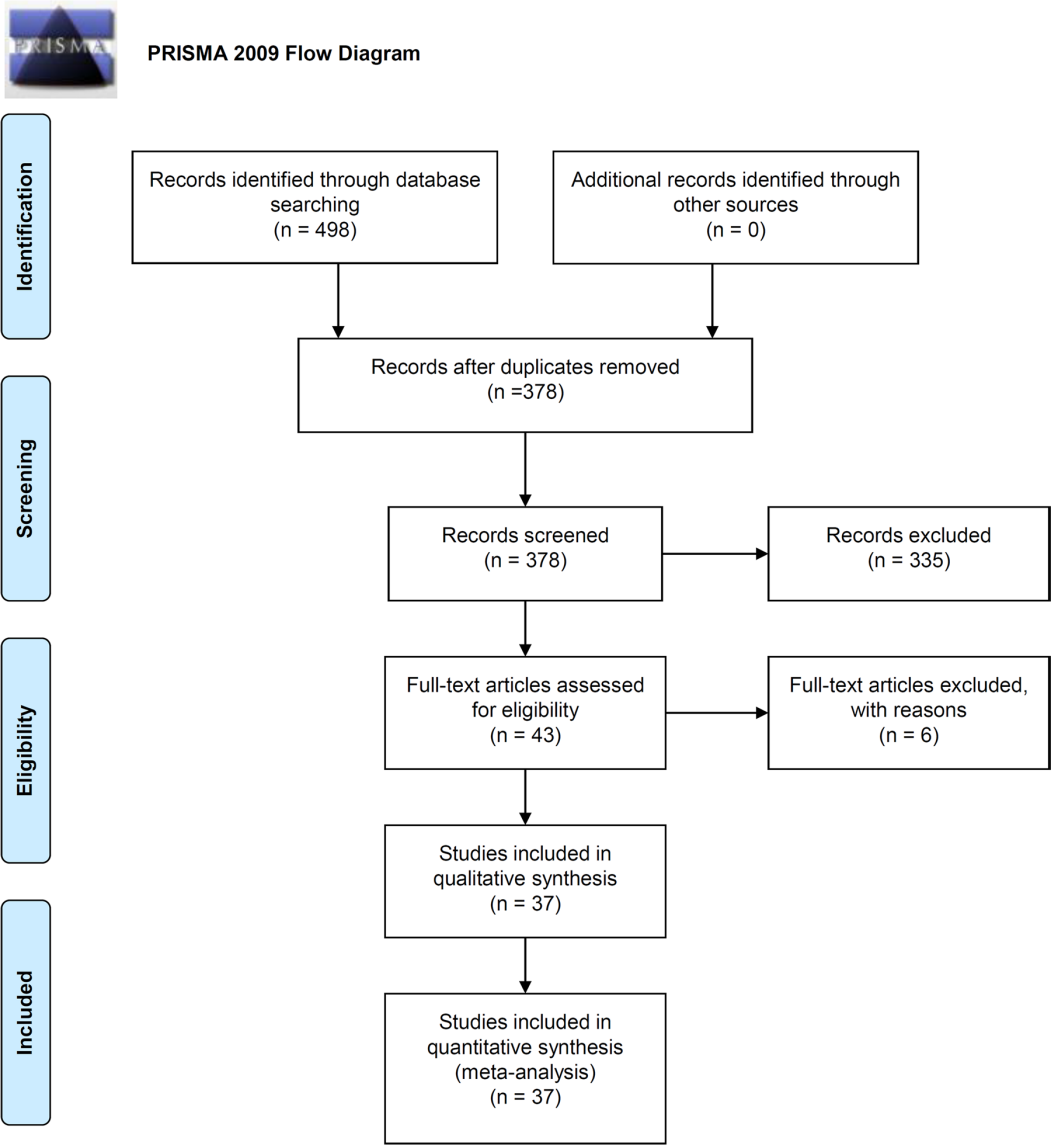
PRISMA 2009 flow diagram showing the process for identifying eligible case-control studies

A flow diagram showing the process by which we identified eligible case-control studies is shown in Figure 1. We initially found 498 articles in the PubMed (*n* = 102), Embase (*n* = 111), and Web of Science (WOS, *n* = 285) databases. We removed 120 duplicate articles, and excluded 179 articles (69 review articles or editorials, 63 case or trial reports, 43 meeting abstract or posters, and four meta-analyses). We also excluded 22 articles that were based on cell or animal data, and 134 articles that involved unrelated diseases, genes, or SNPs. Of the remaining 43 articles, three were excluded due to genotype departure from Hardy-Weinberg equilibrium (HWE) and three due to a lack of available genotype data. We included 37 articles that contained 2, 510 cases and 3,466 controls [6, 11–46] in our meta-analysis. Basic study information is shown in Table 1. All studies had Newcastle-Ottawa Scale (NOS) scores above five.

**Table 1:** Basic information for the studies included in the meta-analysis

First author	Year	Country	Ethnicity	Cases				Disease type	Controls				Assay	Source	NOS
				G/G	G/A	A/A	Total		G/G	G/A	A/A	Total			
Adamczuk	2002	Argentina	Caucasian	37	0	0	37	CRVO	140	4	0	144	PCR-RFLP	PB	8
Albisinni	1998	Italy	Caucasian	32	4*	-	36	RVO	67	1*	-	68	PCR-RFLP	HB	7
Arsene	2005	France	Caucasian	143	10	0	153	CRVO	172	8	0	180	PCR-RFLP	PB/HB	6
			Caucasian	79	2	0	81	BRVO	172	8	0	180	PCR-RFLP	PB/HB	
Batioglu	2003	Turkey	Caucasian	8	7*	-	15	RVO	257	28*	-	285	PCR-RFLP	PB	7
			Caucasian	6	9*	-	15	BRVO	257	28*	-	285	PCR-RFLP	PB	
Biancardi	2007	Brazil	Caucasian	53	2	0	55	RVO	55	0	0	55	PCR-RFLP	HB	6
Bombeli	2002	Switzerland	Caucasian	65	3*	-	68	RVO	112	8*	-	120	PCR-RFLP	PB	7
Ciardella	1998	USA	Caucasian	29	1	0	30	RVO	46	1	0	47	PCR-RFLP	HB	7
Cruciani	2003	Italy	Caucasian	29	0	0	29	RVO	61	1	0	62	PCR-RFLP	PB	7
De Polo	2015	Italy	Caucasian	32	5	0	37	RVO	43	2	0	45	PCR-RFLP	PB	7
Delahousse	1998	France	Caucasian	76	7	0	83	RVO	60	0	0	60	PCR-RFLP	PB	6
Demirci	1999	Turkey	Caucasian	20	3	0	23	CRVO	109	11	0	120	PCR-RFLP	PB	7
			Caucasian	24	1	0	25	BRVO	109	11	0	120	PCR-RFLP	PB	
Di Capua	2010	Italy	Caucasian	109	8	0	117	RVO	191	11	0	202	PCR-RFLP	PB	9
Dixon	2016	USA	Caucasian	52	8	0	60	RVO	60	2	0	62	PCR	PB	7
Dodson	2003	UK	Caucasian	39	1	0	40	RVO	39	1	0	40	PCR-RFLP	PB	9
Faude	1999	Germany	Caucasian	101	6	0	107	CRVO	66	4	0	70	PCR-RFLP	PB	6
Giannaki	2013	Greece	Caucasian	47	4	0	51	RVO	46	5	0	51	CVD Strip Assay	PB	8
Glueck	1999	USA	Caucasian	14	3	0	17	RVO	226	7	0	233	PCR-RFLP	PB	8
Glueck	2005	USA	Caucasian	20	3	0	23	RVO	43	1	0	44	PCR	PB	7
Gori	2004	Italy	Caucasian	99	13	0	112	RVO	107	5	0	112	PCR-RFLP	PB	9
Graham	1996	Australia	Caucasian	22	1	0	23	CRVO	109	4	0	113	PCR-RFLP	PB	7
Greiner	1999	Germany	Caucasian	35	12	1	48	CRVO	32	3	0	35	PCR	HB	5
			Caucasian	27	6	0	33	BRVO	32	3	0	35	PCR	HB	
Horoz	2005	Turkey	Caucasian	29	2	1	32	BRVO	27	3	0	30	NR	PB	8
Johnson	2001	Canada	Caucasian	43	1	0	44	CRVO	68	3	0	71	PCR-RFLP	HB	6
Kalayci	1999	Turkey	Caucasian	48	4	0	52	RVO	75	6	0	81	PCR-RFLP	PB	7
			Caucasian	24	3	0	27	BRVO	75	6	0	81	PCR-RFLP	PB	
Karska-Basta	2013	Poland	Caucasian	53	6	0	59	RVO	50	9	0	59	PCR	PB	8
Koylu	2017	Turkey	Caucasian	43	3	3	49	RVO	64	4	0	68	PCR-RFLP	PB	7
Kuhli	2002	Germany	Caucasian	129	11	2	142	RVO	122	6	0	128	PCR-RFLP	PB	7
Kuhli-Hattenbach	2017	Germany	Caucasian	34	8	0	42	RVO	230	11	0	241	PCR-RFLP	PB	7
Lahey	2002	USA	Mixed	53	2	0	55	CRVO	21	0	0	21	Coatest APC Resistance V Kit	PB	7
Larsson	1997	Sweden	Caucasian	74	8	1	83	CRVO	90	10	1	101	PCR-RFLP	PB	6
Marcucci	2001	Italy	Caucasian	88	12	0	100	CRVO	96	4	0	100	PCR-RFLP	PB	7
Marcucci	2003	Italy	Caucasian	47	8*	-	55	RVO	59	2*	-	61	PCR-RFLP	PB	8
Mrad	2014	Tunisie	African	46	42	0	88	RVO	94	5	0	99	PCR-RFLP	PB	8
Rehak	2010	Czech	Caucasian	74	5*	-	79	CRVO	56	4*	-	60	Allele-specific PCR	HB	7
			Caucasian	36	6*	-	42	BRVO	56	4*	-	60	Allele-specific PCR	HB	
Risse	2014	Germany	Caucasian	83	3	0	86	CRVO	39	1	0	40	PCR	PB	7
			Caucasian	45	2	0	47	BRVO	39	1	0	40	PCR	PB	
Salomon	1998	Israel	Asian	95	7*	-	102	RVO	96	9*	-	105	PCR-RFLP	HB	6
Yioti	2013	Greece	Caucasian	47	1	0	48	RVO	53	0	0	53	CVD Strip Assays	HB	6

RFLP, restriction fragment-length polymorphism; CVD, Cardiovascular disease panel; *, the frequency of the G/A+A/A genotype.

### Overall meta-analysis

**Figure 2:**
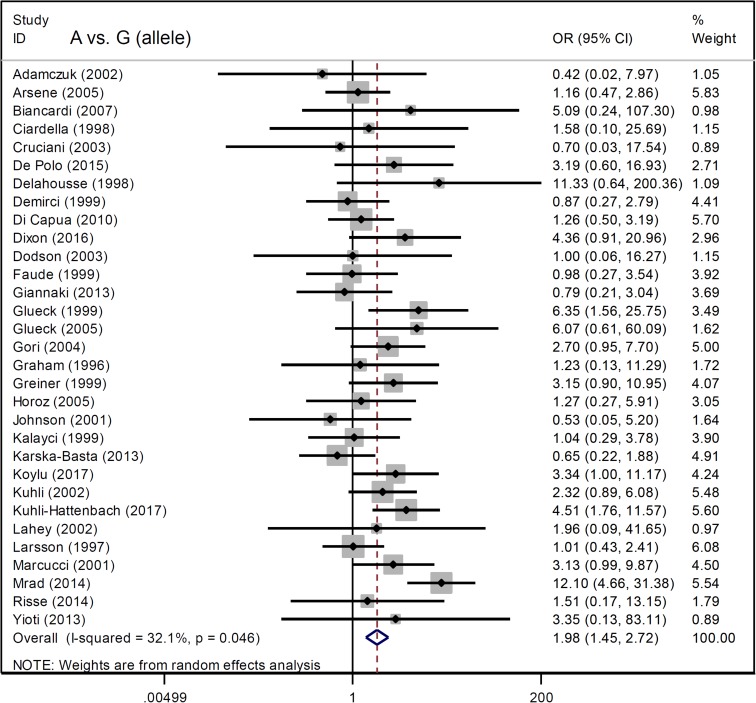
Forest plot data for the meta-analysis under the A vs. G (allele) model

**Figure 3:**
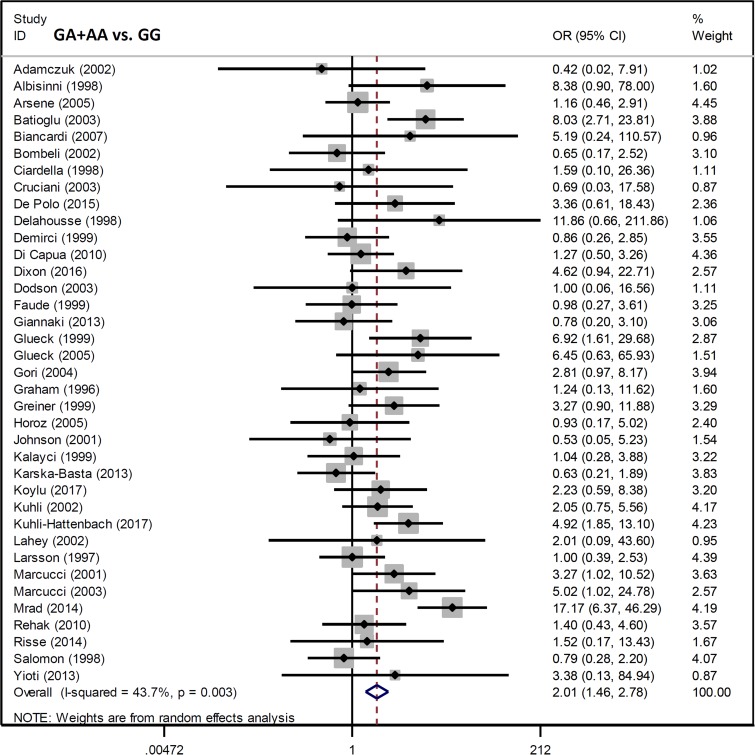
Forest plot data for the meta-analysis under the GA+AA vs. GG model

We analyzed the association between *Factor V* G1691A and RVO susceptibility using a fixed-effects model and Mantel-Haenszel statistics. We did not observe a high degree of heterogeneity between the various models (AA vs. GG, AA vs. GG+GA, A vs. G (carrier) [all I^2^ < 50%, *P* value of the heterogeneity test (*P*_H_) > 0.1] (Table 2). An increased risk of RVO in cases compared to controls was observed under allele, heterozygote, dominant, and carrier models (G vs. A, *P* value of the association test [*P*_A_] < 0.001, odds ratio [OR] = 1.98; GA vs. GG, *P*_A_ < 0.001, OR = 1.90; GA+AA vs. GG, *P*_A_ < 0.001, OR = 2.01; A vs. G carrier, *P*_A_ < 0.001, OR = 1.96), but not homozygote and recessive models (all *P*_A_ > 0.05). Forest plots are shown for the meta-analysis under A vs. G (allele) (Figure 2), GA+AA vs. GG (Figure 3), AA vs. GG (Supplementary Figure 1), GA vs. GG (Supplementary Figure 2), AA vs. GG+GA (Supplementary Figure 3), and A vs. G (carrier) (Supplementary Figure 4) models. These data indicate that the G/A genotype of *Factor V* G1691A is associated with an increased risk of RVO.

**Table 2:** Meta-analysis of the association between Factor V G1691A and RVO

Genetic models	Case-control study number	Sample size	Association test		Heterogeneity test			Begg’s test		Egger’s test	
		Case/control	OR (95% CI)	*P*_A_	I^2^ (%)	*P*_H_	Model	z	*P*_*B*_	t	*P*_E_
**A vs. G (allele)**	31	2,113/2,767	1.98 (1.45∼2.72)	**< 0.001**	32.1%	0.046	Random	0.54	0.587	−0.13	0.897
**AA vs. GG**	5	387/362	3.38 (0.93∼12.35)	0.065	0.0%	0.855	Fixed	-0.24	1.000	0.22	0.843
**GA vs. GG**	31	2,113/2,767	1.90 (1.34∼2.70)	**< 0.001**	39.8%	0.013	Random	0.68	0.497	−0.28	0.784
**GA+AA vs. GG**	37	2,510/3,466	2.01 (1.46∼2.78)	**< 0.001**	43.7%	0.003	Random	0.67	0.505	0.08	0.937
**AA vs. GG+GA**	5	387/362	3.30 (0.90∼12.04)	0.071	0.0%	0.843	Fixed	-0.24	1.000	0.16	0.882
**A vs. G (carrier)**	31	2,113/2,767	1.96 (1.55∼2.48)	**< 0.001**	18.0%	0.189	Fixed	0.61	0.541	−0.09	0.928

*P*_B_, *P* value of Begg’s test; *P*_E_, *P* value of Egger’s test.

### Subgroup meta-analysis

We next performed a subgroup analysis based on ethnicity, source of controls (population-based [PB] or hospital-based [HB]), and disease type (BRVO/CRVO) under all genetic models. *Factor V* G1691A was associated with an increased risk of RVO compared to controls in a Caucasian population under A vs. G (allele) (Table 3, *P*_A_ < 0.001, OR = 1.75), GA vs. GG (*P*_A_ < 0.001, OR = 1.66), GA+AA vs. GG (*P*_A_ < 0.001, OR = 1.88), and A vs. G (carrier) (*P*_A_ < 0.001, OR = 1.66) models. *Factor V* G1691A was also associated with an increased risk of RVO among cases compared to PB controls. Eight BRVO and 13 CRVO studies were included in the disease subgroup meta-analysis. We observed an increased risk of CRVO, but not BRVO, under allele, heterozygote, dominant, and carrier models. Forest plots for the subgroup analysis under the A vs. G (allele) and GA+AA vs. GG models are shown in Supplementary Figures 5–6. Our data indicate G/A genotype of *Factor V* G1691A is associated with an increased risk of RVO (particularly CRVO) in Caucasians.

**Table 3:** Subgroup analysis of the association between Factor V G1691A and RVO

Genetic model	Subgroup	Case-control study number	Association test		Sample size	Heterogeneity test	
			OR (95% CI)	*P*_A_	Case/ Control	I^2^ (%)	*P*_H_
**A vs. G (allele)**	Caucasian	29	1.75 (1.35∼2.28)	**< 0.001**	1,970/2,647	4.0%	0.405
	BRVO	6	1.11 (0.59∼2.08)	0.750	245/486	0.0%	0.719
	CRVO	12	1.66 (1.14∼2.42)	**0.008**	840/1,030	0.0%	0.744
	PB	25	2.03 (1.41∼2.92)	**< 0.001**	1,621/2,326	40.6%	0.019
	HB	5	2.29 (0.91∼5.77)	0.080	258/261	0.0%	0.697
**AA vs. GG**	Caucasian	5	3.38 (0.93∼12.35)	0.065	287/362	0.0%	0.855
	CRVO	3	1.70 (0.30∼9.73)	0.548	212/171	0.0%	0.931
	PB	4	3.90 (0.95∼16.06)	0.060	306/327	0.0%	0.768
**GA vs. GG**	Caucasian	29	1.66 (2.16∼1.28)	**< 0.001**	1,970/2,647	5.5%	0.381
	BRVO	6	1.01 (0.52∼1.95)	0.987	245/486	0.0%	0.643
	CRVO	12	1.65 (1.12∼2.44)	**0.012**	840/1,030	0.0%	0.780
	PB	25	1.93 (1.28∼2.90)	**0.002**	1,621/2,326	48.2%	0.004
	HB	5	2.25 (0.87∼5.79)	0.093	258/261	0.0%	0.703
**GA+AA vs. GG**	Caucasian	34	1.88 (1.42∼2.50)	**< 0.001**	2,265/3,241	22.0%	0.128
	BRVO	8	1.89 (1.15∼3.11)	**0.011**	302/831	60.6%	0.013
	CRVO	13	1.60 (1.11∼2.33)	**0.013**	919/1.090	0.0%	0.761
	PB	28	2.13 (1.45∼3.13)	**< 0.001**	1,759/2,792	50.6%	0.001
	HB	8	1.59 (0.89∼2.84)	0.117	517/494	0.0%	0.440
**AA vs. GG+GA**	Caucasian	5	3.39 (0.90∼12.04)	0.071	287/362	0.0%	0.843
	CRVO	3	1.52 (0.26∼8.74)	0.639	212/171	0.0%	0.958
	PB	4	3.89 (0.94∼16.03)	0.060	306/327	0.0%	0.773
**A vs. G (carrier)**	Caucasian	29	1.66 (1.29∼2.14)	**< 0.001**	1,970/2,647	0.0%	0.632
	BRVO	6	1.05 (0.54∼2.01)	0.892	245/486	0.0%	0.764
	CRVO	12	1.58 (1.07∼2.33)	**0.020**	840/1,030	0.0%	0.860
	PB	25	2.03 (1.57∼2.61)	**< 0.001**	1,621/2,326	28.3%	0.095
	HB	5	2.16 (0.90∼5.20)	0.085	258/261	0.0%	0.743

### Publication bias and sensitivity analysis

**Figure 4:**
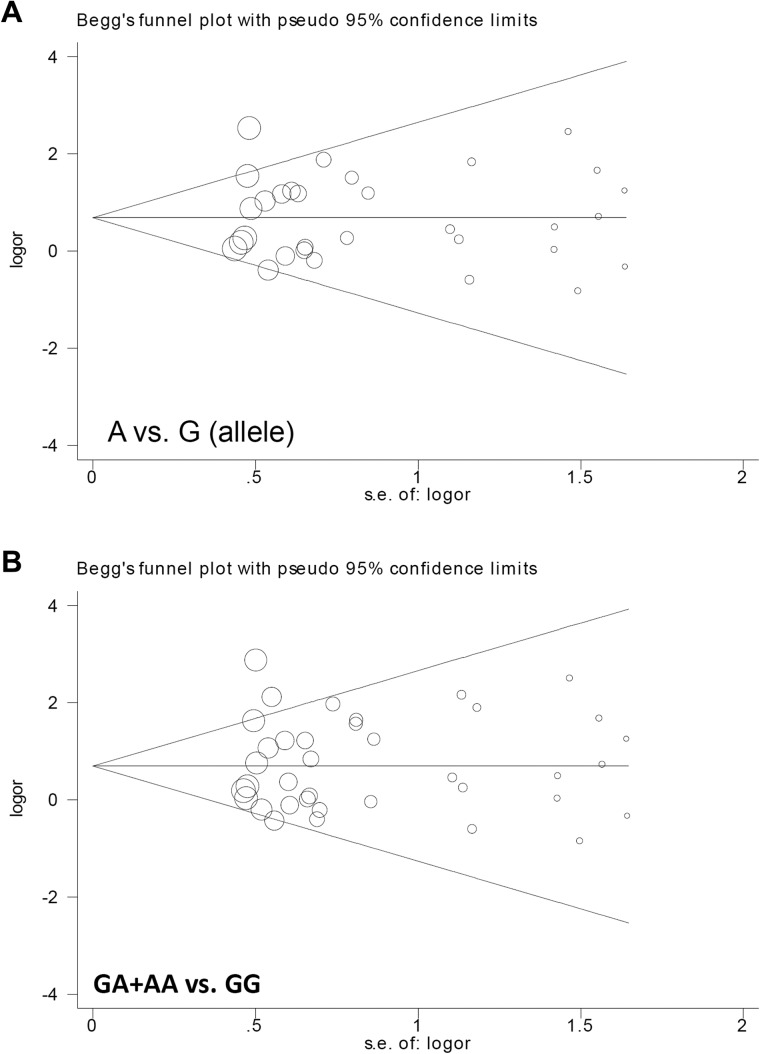
Begg’s funnel plot data with pseudo 95% confidence limits (**A**) A vs. G (allele); (**B**) GA+AA vs. GG.

**Figure 5:**
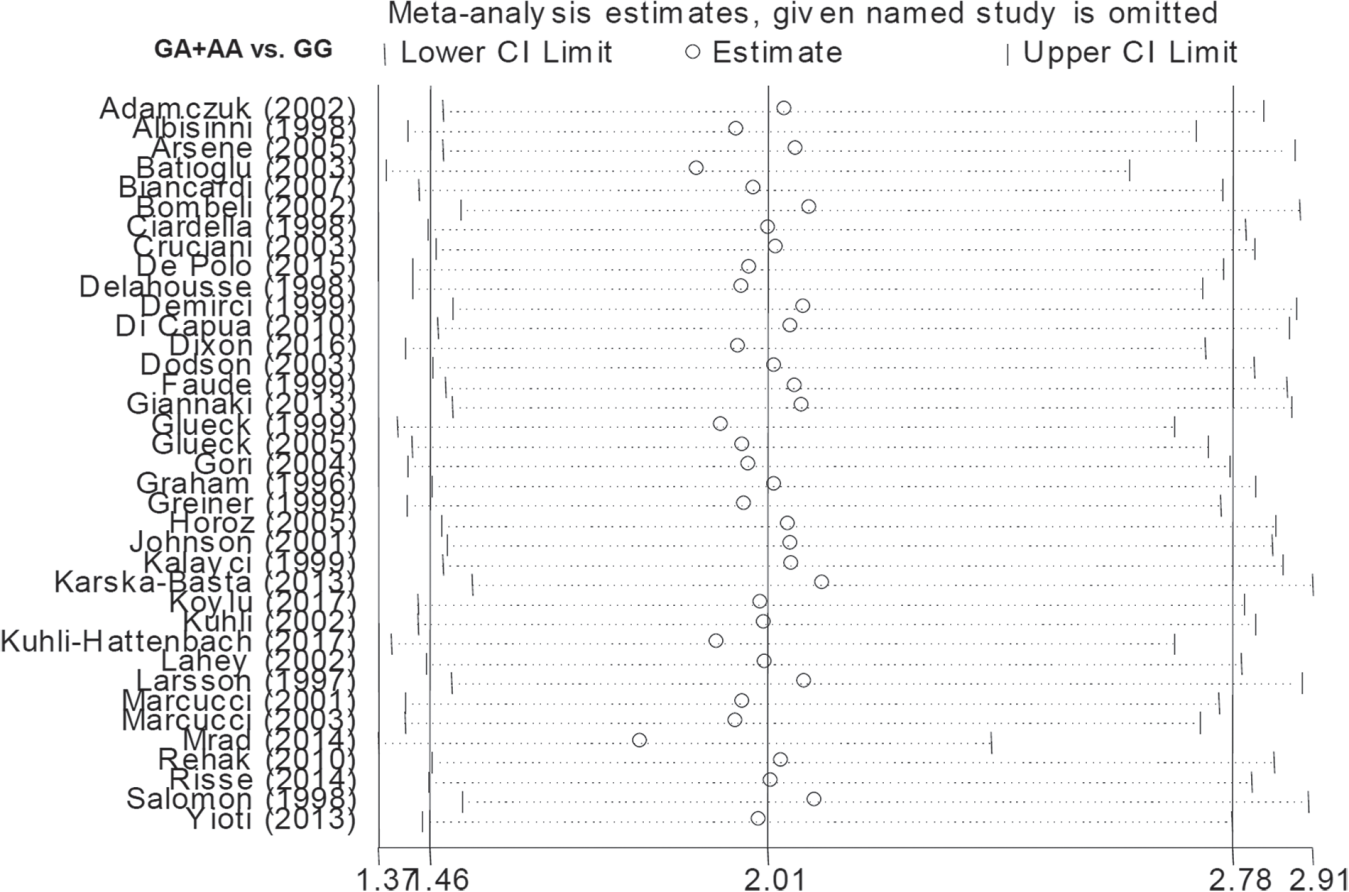
Sensitivity analysis data under the GA+AA vs. GG model

Our analysis indicated there was no publication bias (Table 2, all *P*_Begg_ > 0.05 and *P*_Egger_ > 0.05). Begg’s funnel plots with pseudo 95% confidence limits under the A vs. G (allele) and GA+AA vs. GG models are shown in Figure 4. Sensitivity analysis (Figure 5 for the GA+AA vs. GG model and data not shown) was indicative of stable statistical results.

## DISCUSSION

The G/A genotype of *Factor V* G1691A was previously associated with an increased risk of RVO in French [14] and Tunisian [43] populations. However, no differences in the frequency of the *Factor V* G1691A polymorphism between RVO cases and controls were reported in studies of Turkish populations [22, 32, 36]. Janssen et al. performed a meta-analysis of 12 studies [12, 13, 17, 22, 27, 33, 34, 39, 41, 42, 46, 47] and found that the *Factor V* Leiden mutation (G/A+A/A) was associated with an increased risk of RVO [48]. Rehak et al. performed a meta-analysis of 18 studies [13, 14, 17, 19, 22, 29, 31, 34, 37, 40–42, 47, 49–53] and reported similar results [52]. Finally, Yioti et al. performed a meta-analysis of 21 case-control studies [11–14, 16–19, 21–23, 28, 29, 33, 34, 37, 41, 42, 51, 53, 54] and demonstrated that the *Factor V* Leiden mutation was associated with an increased risk of RVO [11].

The A/A genotype of *Factor V* was previously observed in several studies [31, 32, 36, 37, 40]. However, only the contribution of G/A+A/A genotype of *Factor V* G1691A to RVO was described; the roles of the individual G/A or A/A genotypes were not investigated. Several factors including ethnic background, source of controls (PB/HB), and disease type (BRVO/CRVO) were also not sufficiently analyzed in previous studies [11, 48, 52]. Therefore, we performed a meta-analysis of 37 case-control studies, under allele (A vs. G), homozygote (AA vs. GG), heterozygote (GA vs. GG), dominant (GA+AA vs. GG), recessive (AA vs. GG+GA), and carrier (A vs. G) models. Our data indicate that the presence of a single *Factor V* Leiden allele increases the risk of RVO. The G/A genotype of *Factor V*, but not the A/A genotype, was an inherited risk factor for RVO in a Caucasian population. Moreover, when we stratified by type of RVO, heterozygosity was associated with an increased risk of CRVO, but not BRVO. The mechanisms underlying the association between *Factor V* G1691A and RVO have not been elucidated. It is possible that *Factor V* mutations lead to resistance to anticoagulant processing, and activated APC resistance or protein S deficiency may be the key points, which are worthy of future experiment data.

Our study had several advantages. First, we performed a quantitative analysis of a large number of case-control studies selected from three independent databases. Second, we excluded studies involving genotype data that deviated from Hardy-Weinberg Equilibrium, which confirmed the balance of gene frequency and genotype frequency, and enabled rigorous statistical analysis. Third, under the guideline of our strict inclusion and exclusion criteria, the enrolled case-control studies exhibit the high publication quality. Among them, we found that population-based control data is involved in most of studies. The data from the comparison between RVO case and healthy control subjects from the normal population is more helpful to drive a more reasonable statistical assessment for the genetic role of *Factor V* Leiden allele in the clinical RVO cases. We also performed subgroup analyses according to ethnicity (Caucasian/Asian), source of controls (PB/HB), and disease type (BRVO/CRVO). Finally, we detected no publication bias and demonstrated stable statistical results in a sensitivity analysis.

Our study also had several disadvantages. First, the sample size of the included case-control studies was relatively small, which limited the statistical power in the subgroup meta-analysis. For example, only one case-control study was enrolled in the subgroup analysis for the association between *Factor V* G1691A and susceptibility to RVO in an Asian population [46]. Second, although there was no clear association between the A/A genotype of *Factor V* G1691A and the risk of RVO, we cannot exclude the potential genetic effect of homozygosity. The low prevalence of the A/A genotype may have contributed to the underpowered meta-analysis. Third, only the G1691A SNP was investigated in our study due to data availability. We did not analyze the role of other SNPs (e.g. G4070A), or the combination of *Factor V* and other relative genes such as *MTHFR* and *prothrombin*. Fourth, the main clinical types of retinal vein occlusion, namely BRVO and CRVO, and other uncommon types, such as bilateral RVO, exhibit the differences or complexity of physiopathology [1–3, 55]. Unfortunately, we failed to obtain the SNP data of the association between *Factor V* Leiden and bilateral RVO risk. Confounding factors such as sex, age of onset, family history, lifestyle, clinical type, and complications should be investigated in future meta-analyses of a larger number of subjects with different types of RVO. Finally, heterogeneity was observed between the A vs. G (allele), GA vs. GG, GA+AA vs. GG genetic models, which could have biased our analysis. However, no heterogeneity was observed in the subgroup analysis of Caucasians (all I^2^ < 50%, *P*_H_ > 0.1). Similarly, we observed no heterogeneity between the BRVO/CRVO subgroups, with the exception of the BRVO subgroup under the GA+AA vs. GG models. Thus, ethnicity and disease type may have contributed to the observed heterogeneity. Our meta-analysis indicates that the G/A genotype of *Factor V* G1691A is associated with an increased risk of RVO, particularly CRVO, in Caucasians.

## MATERIALS AND METHODS

### Database retrieval and article screening

Using the guidelines of the “Preferred Reporting Items for Systematic Reviews and Meta-Analyses (PRISMA)” [56], we retrieved articles published before July 27, 2017 from the PubMed, Embase, and WOS databases. Search terms for PubMed included the following: ((((((((((Retinal Vein Occlusion[MeSH Terms]) OR occlusion, retinal vein) OR occlusions, retinal vein) OR retinal vein thrombosis) OR retinal vein thromboses) OR thromboses, retinal vein) OR vein thromboses, retinal) OR vein thrombosis, retinal) OR thrombosis, retinal vein)) AND (((((((((*Factor V* [Other Term]) OR Proaccelerin) OR AC Globulin) OR Coagulation *Factor V*) OR *Factor V*, Coagulation) OR Factor Pi) OR Blood Coagulation *Factor V*) OR FV Leiden) OR *Factor V* G1691A). We excluded duplicate articles, and then screened and removed ineligible articles using the following exclusion criteria: (1) review article or editorial, (2) case or trial report, (3) meeting abstract or poster, (4) meta-analysis, (5) cell- or animal-based study, (6) unrelated disease, gene, or SNP (7) departure from HWE, and (8) lack of available genotype data.

### Data extraction and NOS assessment

Three authors independently extracted data from eligible articles including the name of the first author, publication year, country, ethnicity of the study population, genotype frequencies, disease type, genotyping assay, study number, sample size of case/control populations, and source of controls. We assessed the methodological quality of eligible studies using the NOS system (http://www.ohri.ca/programs/clinical_epidemiology/oxford.asp).

### Statistical analysis

Mantel-Haenszel statistical analysis under fixed- or random-effect models was performed with the Stata/SE 12.0 software (StataCorp, USA). A fixed-effect model was utilized where Cochran’s Q statistic (*P*_H_) > 0.1 or I^2^ < 50 %. ORs, 95% CIs, and *P*_A_ values were calculated in allele (A vs. G), homozygote (AA vs. GG), heterozygote (GA vs. GG), dominant (GA+AA vs. GG), recessive (AA vs. GG+GA), and carrier (A vs. G) models. Subgroup analysis was performed according to ethnicity, source of controls (PB/HB), and disease type (BRVO/CRVO) under all genetic models. Publication bias was evaluated using Begg’s and Egger’s tests and sensitivity analysis was performed.

## SUPPLEMENTARY MATERIALS FIGURES


