# Supplementary material for: Factor V G1691A is associated with an increased risk of retinal vein occlusion: a meta-analysis
Source: Oncotarget. 2017 Sep 4;8(43):75467–77. doi: 10.18632/oncotarget.20636 (PMC5650437; doi:10.18632/oncotarget.20636)
Supplement: Supplementary file 1 [file oncotarget-08-75467-s001.pdf]

Factor V G1691A is associated with an increased risk of retinal vein occlusion: a meta-analysis

SUPPLEMENTARY MATERIALS

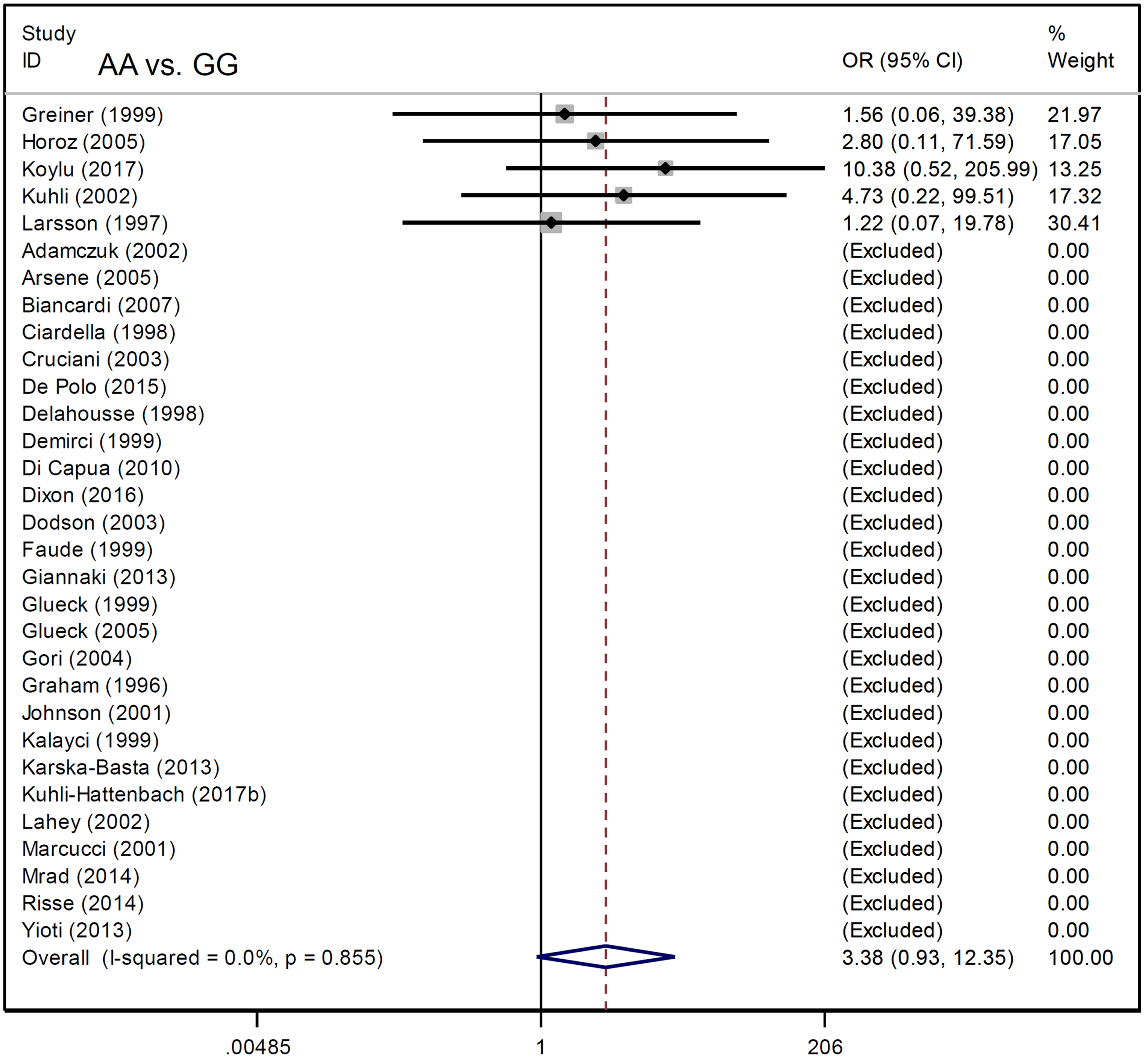

Supplementary Figure 1: Forest plot data for the meta-analysis under the AA vs. GG model.

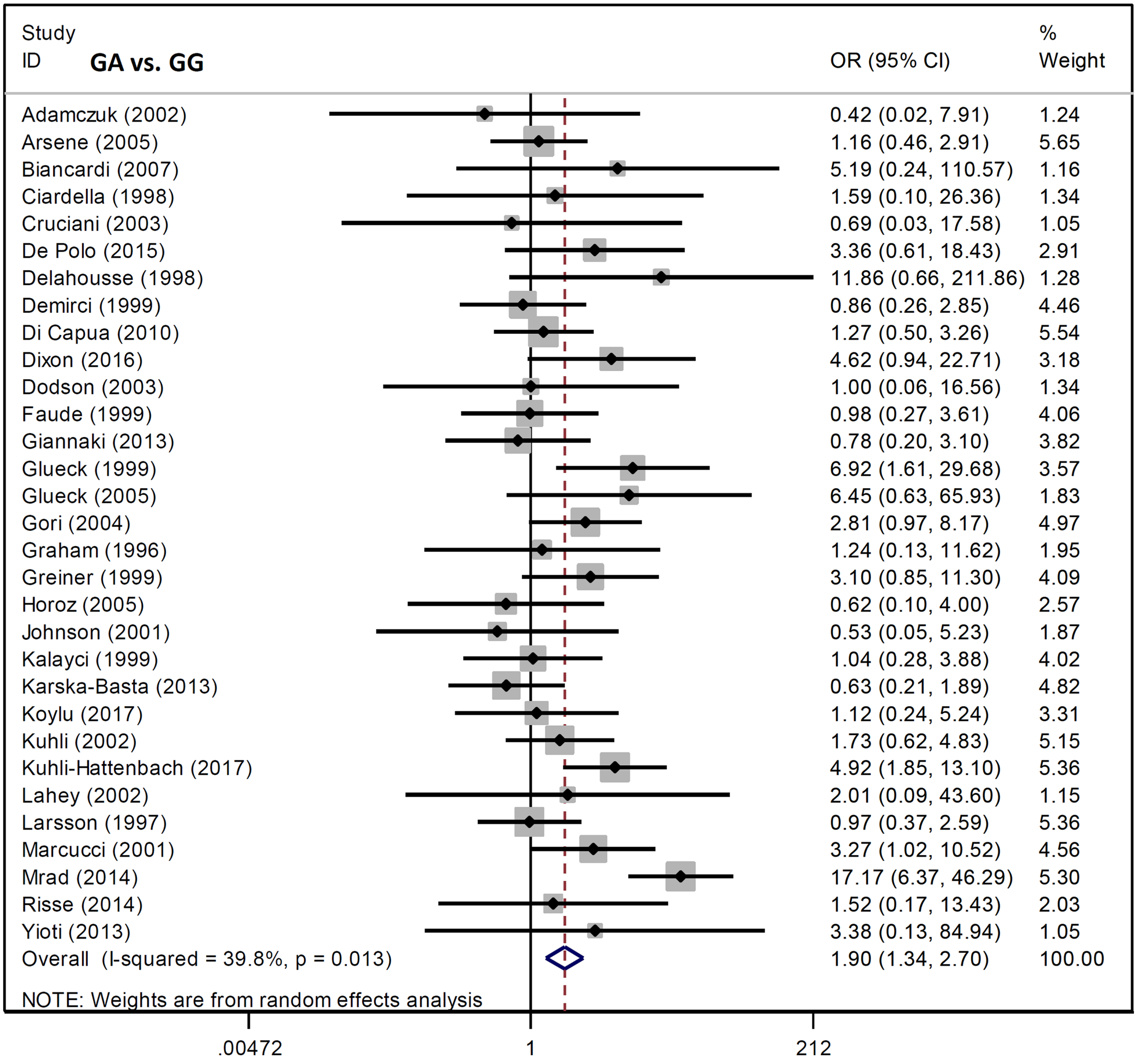

Supplementary Figure 2: Forest plot data for the meta-analysis under the GA vs. GG model.

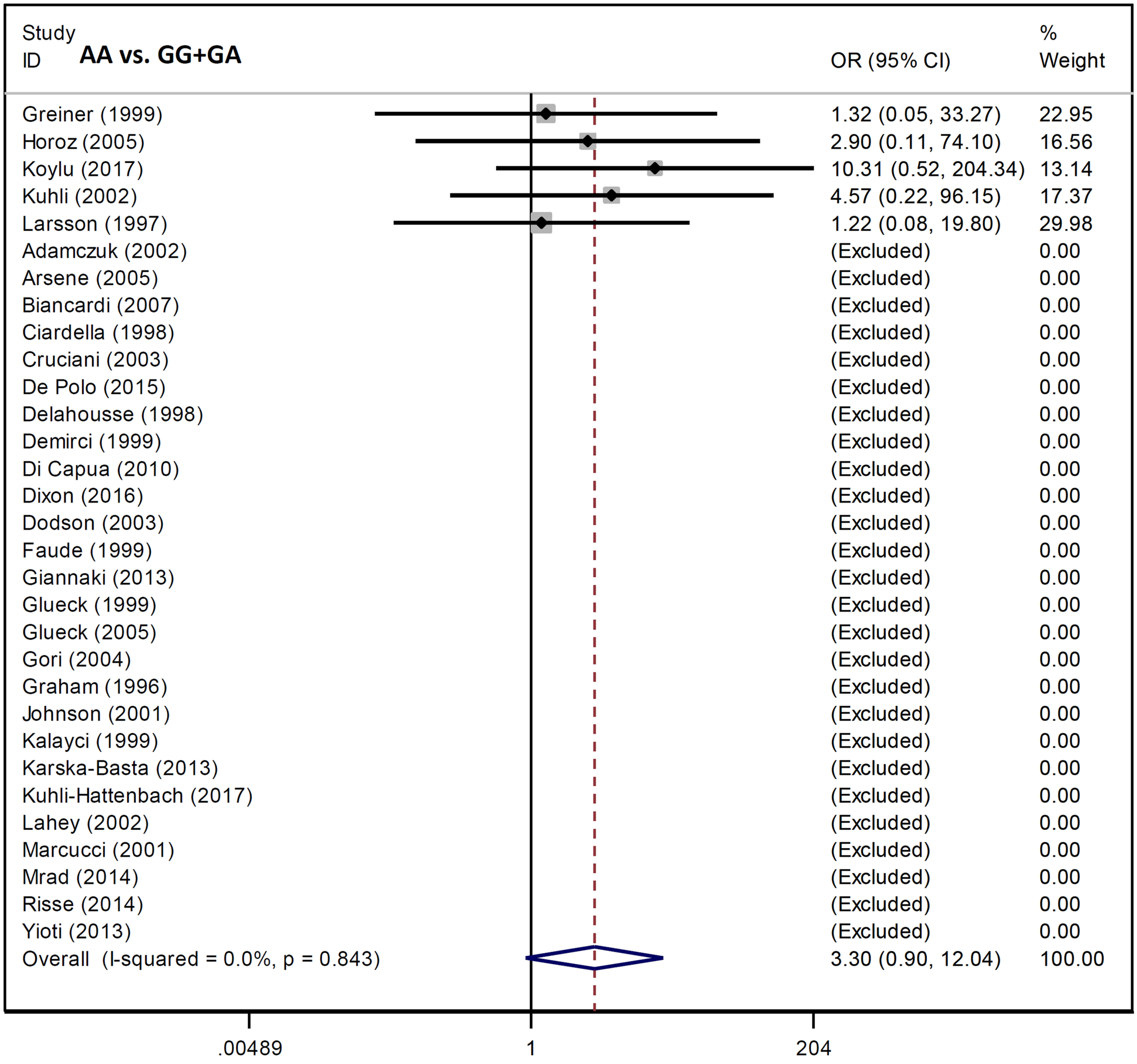

Supplementary Figure 3: Forest plot data for the meta-analysis under the AA vs. GG+GA model.

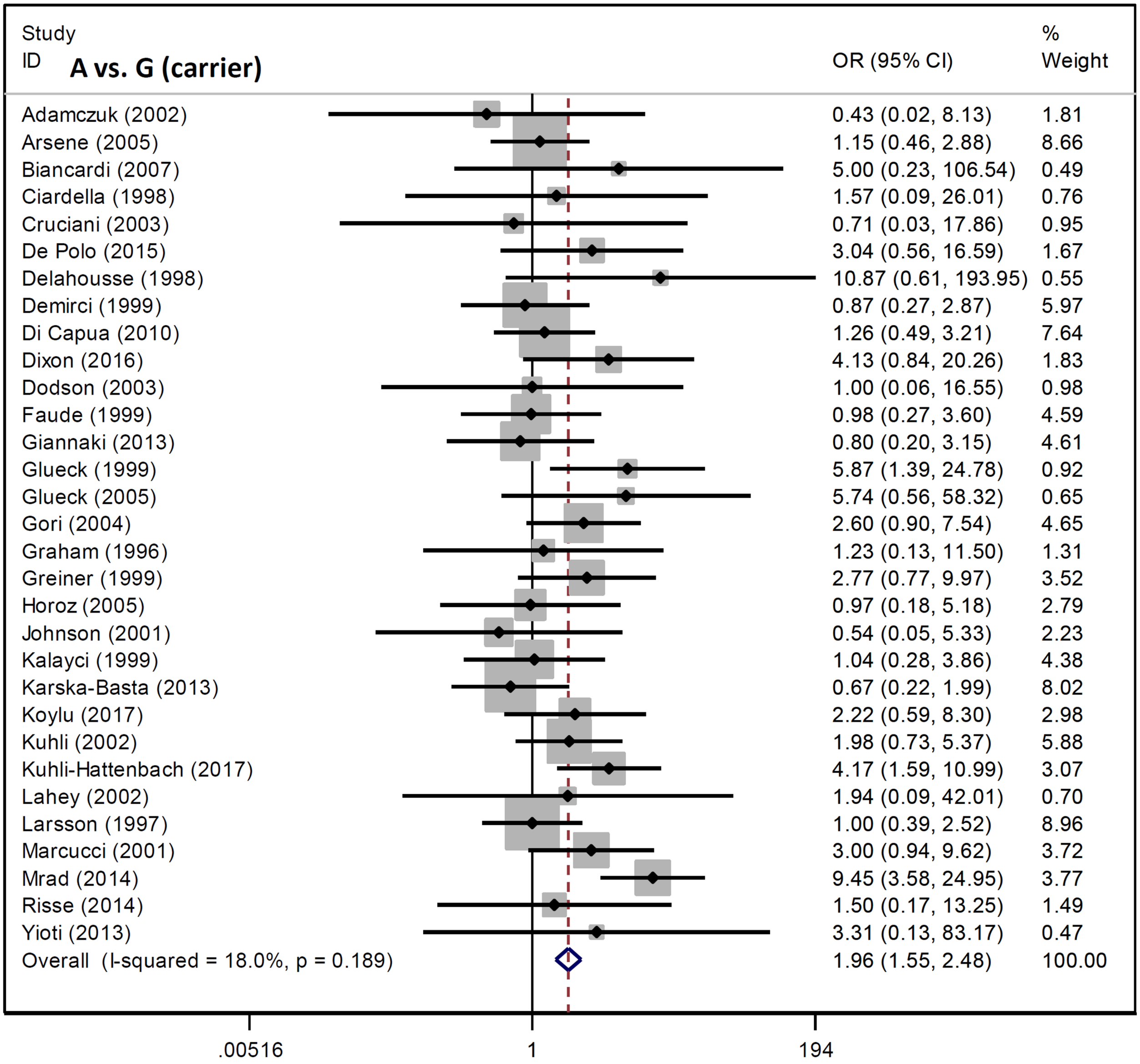

Supplementary Figure 4: Forest plot data for the meta-analysis under the A vs. G (carrier) model.

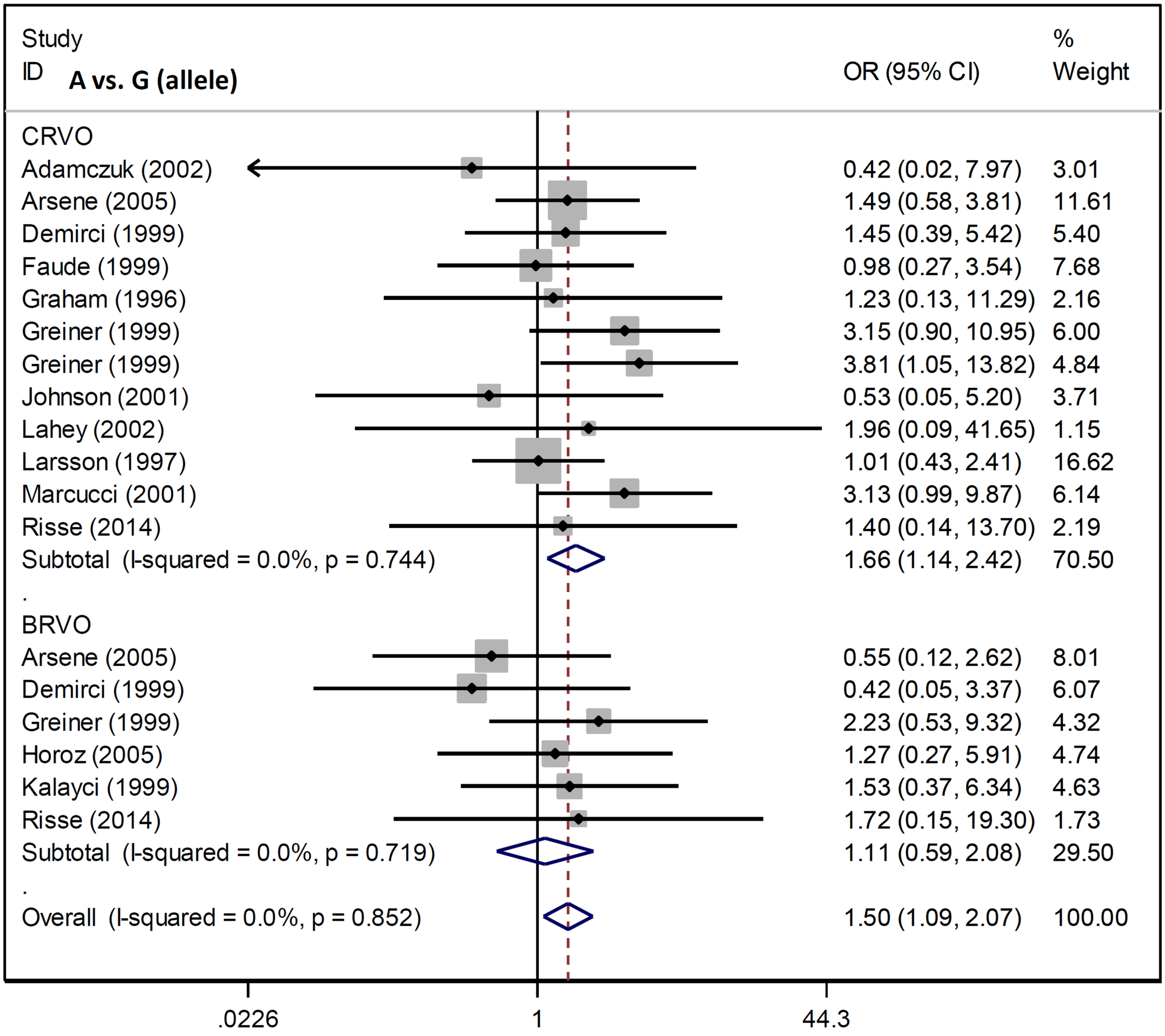

Supplementary Figure 5: Subgroup analysis according to RVO type under the A vs. G (allele) model.

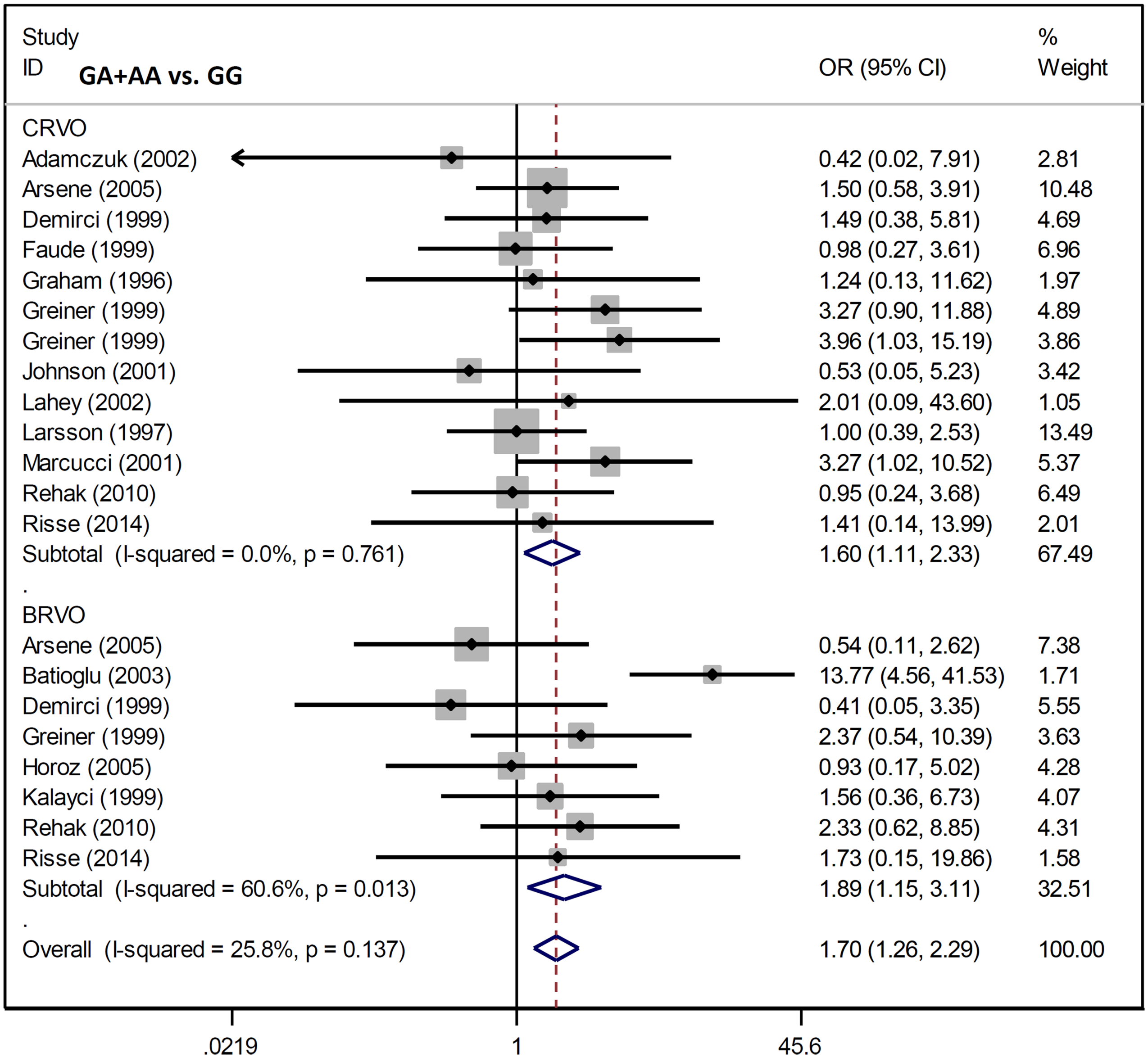

Supplementary Figure 6: Subgroup analysis according to RVO type under the GA+AA vs. GG model.
